# New Methods in the Treatment of Cholera

**Published:** 1885-11

**Authors:** A. Lagorio

**Affiliations:** Chiavari


### FOREIGN CORRESPONDENCE.

---

LAGORIO. NEW METHODS IN THE TREATMENT OF CHOLERA.

*To the Editors of the* CHICAGO MEDICAL JOURNAL AND EXAMINER.

*Gentlemen:*

In some of my former communications I had occasion to speak of hypodermoclisis (subcutaneous injection of warm, salted water), which was largely made use of during the cholera epidemic of last year throughout the kingdom of Italy. I have been for some time anxiously awaiting a report, as to its real efficacy, the indications for its use, and how and when it was to be used. At last it has appeared, and a valuable one it is, being from the pen of the originator, the illustrious PROFESSOR ARNALDO CANTANI, of Naples (*Morgagni*, July, 1885). Since 1865, the Professor has proposed this operation in the treatment of cholera, for it replaces in the system the amount of fluid lost through the intestinal discharges, which are one of the most important causes of death.

He gives the history of 226 cases of Asiatic cholera treated with his method; of these, 155 recovered, and 71 died. If we consider that the hypodermoclisis was only performed in the worst and most desperate cases, and that it gave such a large percentage of cures, we must look upon it as a useful curative means. The operation has met, especially in Naples, great opposition, but generally from physicians who had practiced it in an imperfect way, and therefore with negative results. PROFESSOR MARINI did it in 101 cases, with 92 recoveries, and only 9 deaths, which occurred in those cases in which the stage of collapse had reached the highest degree. DR. POGGI used it in 15 desperate cases, with 9 recoveries, 7 of which were in an advanced stage of collapse. PROFESSOR MARAGLIANO, of Genoa, also speaks of it with great praise. The operation consists in injecting slowly, with a suitable apparatus, into the lateral regions of the abdomen, the following solution : chloride of sodium, 4 grams (ʒi), carbonate of sodium, 3 grams (gr. 45), water, one liter, previously warmed to 38 or 40 degs. Cent. When the patient has reached the cold stage of asphyxia, the amount of liquid to be injected at one time must never exceed 1,500 c. c. ; 800 to 1,000 c. c. are sufficient in most cases ; 500 to 600 c. c., when the patient is in a typhoid state. It is indispensable that in most cases the operation be repeated *several times* at brief intervals, until the pulse becomes stronger, respiration better, cyanosis diminished, and the urine reappears. The intervals are to be shorter, especially in the typhoid state.

Besides hypodermoclisis, another operation called enteroclisis (injections of medicated warm solutions into the intestines per rectum) also gave very brilliant results. As CANTANI has already written (*Morgagni*, 1879), the direct treatment of the intestine, which is the primary and principal region affected in cholera, is the most rational, especially in the beginning of the choleraic infection, so as to be able to abort the disease. The Professor has devised and introduced in practice an apparatus, already well-known in the kingdom as Cantani's enteroclimus, which has been proven to be able to pass the ileo-cæcal valve and inject the liquid into the small intestines by an anti-peristaltic movement that this method excites. To disinfect the locality of the disease, which is the seat of the choleraic microphyte, many substances may be used; all the acids capable of rendering the intestinal contents acid· Muriatic, nitric, phosphoric, sulphuric acids may all be theoretically useful, not in killing the choleraic microphyte in the doses in which we are permitted to introduce these acids into the intestine, but to depress the vitality, diminish the power of multiplying, and the vegetative activity. We have also anti-parasitic disinfectants, as carbolic acid, salicylic and boracic acids, sulphurous acid, thymol, resorcine, etc., etc.; also perchloride, sulphate of iron, nitrate of silver, tannic acid, etc. Above all, *corrosive sublimate*, a most potent poison to vegetable life and parasiticide to the comma-bacillus in a proportion which permits its introduction by enteroclisis (1–2 centigr. in two quarts of water). CANTANI has experimented with tannic acid and was highly pleased with it. This remedy is an excellent astringent and tonic to the relaxed vessels, is also a good sterilizer and prevents the multiplication of the bacillus, although it does not kill it.

Enteroclisis was also adopted to *excite* and *warm* the organism, and was, therefore, made *very warm*, 39° to 40° C. If an enteroclisis of two quarts of *cold water*, repeated several times a day, has proven to be the best cooling agent for a typhoid fever patient (by far preferable to the cold bath, for it does not cause such a violent reaction); an *enteroclisis of two quarts*

*of warm water* is also the best warming agent for a patient with sub-normal temperature, and is the *best stimulant* for an intestine which is beginning to be paralyzed and to lose its power of absorption. It is an internal warm bath without the inconveniences of an external warm bath. It has shown its power to prevent anuria, and the excessive thickening of the blood and draining of tissues, and, therefore, it *keeps* and *restores* to the intestine the power to absorb water, which is very important in Asiatic cholera. From all this we can easily understand that when employed in the beginning of the disease, it may abort the attack of cholera, and when repeated many times a day may prevent the stage of asphyxia. In cases where diarrhœa continues, the warm tannic acid enteroclisis is to precede the hypodermoclisis. The quantity of liquid ought to be 1 ½ to 2 quarts of water with 3–6 grams of tannic acid (as high as 10 grams, according to Maragliano) to each quart of water, of infusion of chamomile flowers or of walnut leaves, warmed to 40° C. Sometimes both operations are made at the same time, if the diarrhœa is persistent, and the collapse of the patient lasts. Prof. Cantani admits two stages of cholera: first, of *infection;* second, of *intoxication.* The comma-bacillus acts directly in the first stage; after this the incubation of few days, and the intestinal irritation with diarrhœa and vomiting, and hence the draining of tissues by the rapid loss of serum.

In the second stage there is acting a *ptomainic poison* which may be produced in the tissues themselves by the chemical necrobiolysis of many histological elements, broken up by the suspension of the material change, representing, therefore, a *cadaveric ptomaine* produced during life (ante-mortem),—and may also be produced by the comma-bacillus itself, by the process of fermentation which it undergoes in the contents of the intestine, and becoming absorbed in the blood gives in the manner a *specific ptomaine* of cholera. In both cases the poisoning of the nervous centres and of the sympathetic and progressive paralysis of the heart are caused by the presence of the ptomainic poison in the blood. In lighter cases *infection* is the cause, while in the worst cases there is an addition of ptomaines more or less poisonous, more or less abundant according to the conditions of the intestinal contents encountered by the bacillus. In the rapid (fulminant) cases the development of pernicious ptomaines may be so rapid and great in quantity or quality, that intoxication prevails over infection, so that it may produce its paralyzing effects before the latter can irritate extensively.

In considering the causes of death by cholera, the main factor is certainly the lowering and final arrest of the circulation, and the progressive paresis and paralysis of the heart. A progressive thickening of the blood is a result, caused by the great loss of liquid in the diarrhœa and vomiting. This thickening of the blood so disturbs the nutrition of the entire organism, as to stop the material changes and to render impossible the elimination of the products of consumption by the histological elements of the different organs and tissues.

At first the heart endeavors to conquer the increasing obstacle, which thick blood always opposes; the contractions become more frequent, but finally they cannot overcome it, and the blood stagnates at the periphery, causing cyanosis and a lowering of the peripheric temperature; the stasis increases from the periphery towards the center of the circulation; finally the force of the myocardium is not sufficient to empty the ventricles, and the heart stops.—It is evident that to combat this cause of cardiac paralysis and of death, the only rational means is to restore to the blood the lost fluid, which, if done by the warm alkaline salted hypodermocilisis, which re-establishes the fluidity of the blood, the material changes in all the organs, and in the heart itself, and facilitates the elimination of retained excrementitious substances which poison the organism; moreover, the injected water excites by its warmth the depressed nervous centers, and dilutes the poison concentrated in the blood, provided it is made in time to be absorbed.

The enteroclisis of large quantity of liquid is another rational means of combating the development of the ptomaines themselves in the fœcal matter of the intestines, it promotes the elimination from the intestine of ptomainiferous and fermenting masses, and stops, therefore, the absorption of these poisonous ptomaines; besides, by using disinfectants, anti-fermentatives and antidotes, we can stop the vegetative activity, the ferment of the bacilli in the intestines, and the further production of ptomaines, which we can possibly render innocuous by altering them, destroy or transform them in combinations less easily absorbed or less poisonous. Another cause of death is certainly a diminished alkalinity of the blood. This we remedy by adding to the liquid the alkaline salts of sodium.

I can bear testimony to the usefulness of the warm tannic acid enteroclisis, so much used by Prof. Maragliano, of Genoa, during the epidemic of September and October of last year. It was always the first thing to be done to almost every cholera-stricken patient, and many lives were saved by it. In many the attack was shortened, in others it helped to bring them to a successful cure. Stimulants and sedatives were also used as needed. The percentage of deaths only reach 30 per cent.

Cantani has also proposed to *combat the cholera bacillus in the intestines with other bacteria hostile to it, but not harmful to the man.* At present he has mentioned the *Bacterium termo*, which possesses the two properties of destroying the cultures of the comma-bacillus of Koch without harming in any way the man, and without causing any inconvenience in the intestines. This he has proven with the enteroclisis in sound individuals. Possibly other bacteria are equally useful. Considering that the ordinary chemical disinfectants are generally more dangerous to man than to the parasites, if used in the doses required to destroy the pathogenic microphytes, and considering, also, that the great law of nature presents life to us as a struggle for existence, in which the feebler succumbs to the stronger, and this triumphs at the death of the other, we ought to investigate all the microbes hostile among themselves, but innocuous to the organism of man, and the ones we can place against the pathogenic ones which cause a specific infection, above all if the seat of the infecting bacterium is easily accessible, as in intestine in case of cholera, typhoid fever, dysentery: as in the genital tracts (gonorrhœa, etc.); and as in the respiratory canals and in the fauces (pertussis, diphtheria, etc.). It may be a microphyte will be useful against several other pathogenic ones, or probably some infection will not be fought except with a special microphyte, as small-pox with vaccination, . . . with a view entirely different from that of JENNER and PASTEUR, who want to extinguish the susceptibility to an infection with the attenuation of the same virus. In many cases this bacterio-therapy, this biologic treatment, prophylaxis and hygiene seems certain to have a great future, and is worthy the attention of all colleagues who are sincerely scientific and honest physicians.

A. LAGORIO.

*Chiavari, Oct. 2, 1885.*

